# Quiz-Compends

**Published:** 1903-10

**Authors:** 


Quiz-Compends—Anatomy (No. 1).—By Saml. O. L. Potter, M. A., M. D., M. R. C. P., London. Seventh edition, revised and enlarged. One hundred and thirty-eight wood engravings. Sixteen plates of arteries and nerves.

A Compend of Diseases of the Skin (No. 16).—By Jay F. Schamberg, A. B., M. D. Third edition, revised and enlarged. One hundred and six illustrations. Both published by P. Blakiston's Son & Co., 1012 Walnut Street, Philadelphia. Price of each, 80c net.

These little volumes contain a brief but thorough outline of the subjects discussed and are invaluable for hasty review. The series is too well known to need discussion at our hands, but suffice it to say, to the student preparing for a medical examination, or to the practitioner who desires to make a thorough but hasty review of dermatology or anatomy, these books are of material assistance.

